# The prevalence of thyroid dysfunction and hyperprolactinemia in women with PCOS

**DOI:** 10.3389/fendo.2023.1245106

**Published:** 2023-10-03

**Authors:** Kim van der Ham, Karlijn J. Stekelenburg, Yvonne V. Louwers, Wendy van Dorp, Marco W. J. Schreurs, Ronald van der Wal, Régine P. M. Steegers-Theunissen, Joop S. E. Laven

**Affiliations:** ^1^ Division of Reproductive Endocrinology and Infertility, Department of Obstetrics and Gynecology, Erasmus University Medical Centre, Rotterdam, Netherlands; ^2^ Department of Immunology, Laboratory Medical Immunology, Erasmus University Medical Centre, Rotterdam, Netherlands; ^3^ Department of Internal Medicine, Erasmus University Medical Centre, Rotterdam, Netherlands; ^4^ Department of Obstetrics and Gynecology, Erasmus University Medical Centre, Rotterdam, Netherlands

**Keywords:** PCOS, hyperprolactinemia, reproductive disorders, thyroid dysfunction, TPOab

## Abstract

**Introduction:**

Ovulatory dysfunction is usually caused by an endocrine disorder, of which polycystic ovary syndrome (PCOS) is the most common cause. PCOS is usually associated with estrogen levels within the normal range and can be characterized by oligo-/anovulation resulting in decreased progesterone levels. It is suggested that decreased progesterone levels may lead to more autoimmune diseases in women with PCOS. In addition, it is often claimed that there is an association between hyperprolactinemia and PCOS. In this large well-phenotyped cohort of women with PCOS, we have studied the prevalence of thyroid dysfunction and hyperprolactinemia compared to controls, and compared this between the four PCOS phenotypes.

**Methods:**

This retrospective cross-sectional study contains data of 1429 women with PCOS and 299 women without PCOS. Main outcome measures included thyroid stimulating hormone (TSH), Free Thyroxine (FT4), and anti-thyroid peroxidase antibodies (TPOab) levels in serum, the prevalence of thyroid diseases and hyperprolactinemia.

**Results:**

The prevalence of thyroid disease in PCOS women was similar to that of controls (1.9% versus 2.7%; P = 0.39 for hypothyroidism and 0.5% versus 0%; P = 0.99 for hyperthyroidism). TSH levels were also similar (1.55 mIU/L versus 1.48 mIU/L; P = 0.54). FT4 levels were slightly elevated in the PCOS group, although within the normal range (18.1 pmol/L versus 17.7 pmol/L; P < 0.05). The prevalence of positive TPOab was similar in both groups (5.7% versus 8.7%; P = 0.12). The prevalence of hyperprolactinemia was similarly not increased in women with PCOS (1.3%% versus 3%; P = 0.05). In a subanalysis of 235 women with PCOS and 235 age- and BMI-matched controls, we found no differences in thyroid dysfunction or hyperprolactinemia. In according to differences between PCOS phenotypes, only the prevalence of subclinical hypothyroidism was significantly higher in phenotype B (6.3%, n = 6) compared to the other phenotypes.

**Conclusion:**

Women with PCOS do not suffer from thyroid dysfunction more often than controls. Also, the prevalence of positive TPOab, being a marker for future risk of thyroid pathology, was similar in both groups. Furthermore, the prevalence of hyperprolactinemia was similar in women with PCOS compared to controls.

## Introduction

An irregular or absent menstrual cycle, also known as ovulatory dysfunction, is a common cause of subfertility in women of reproductive age. Ovulatory dysfunction is usually caused by an endocrine disorder, of which polycystic ovary syndrome (PCOS) is the most common cause. The prevalence of PCOS has been estimated at 4-21%, according to the Rotterdam Criteria (1). Along with the reproductive problems PCOS is also associated with increased risk of cardiovascular risk factors, such as obesity, type II diabetes, hypertension, and dyslipidemia (2).

Additional development of autoimmune thyroid disease could aggravate these cardiovascular risk factors. Unfavorably, some studies observed that autoimmune thyroid diseases occur more often in women with PCOS (3). The study of Janssen et al. shows even a threefold higher prevalence of auto immune thyroiditis in these women (4). Estrogen has a stimulating effect on autoimmune diseases (5), whereas progesterone has a protective role as a natural immune suppressor (6). As stated, PCOS is usually associated with estrogenic levels within the normal range and can be characterized by oligo-/anovulation resulting in decreased exposure to progesterone (7). It is suggested that reduced progesterone may lead to more autoimmune diseases in women with PCOS (7, 8). In autoimmune thyroiditis, autoantibodies are formed against one or more components of the thyroid gland. One of these anti-thyroid antibodies are anti-thyroid peroxidase antibodies (TPOab). TPOab have been associated with Hashimoto thyroiditis and atrophic thyroiditis. The appearance of TPOab usually precedes the development of actual thyroid dysfunction and disease and thereby serve as a predictive marker of thyroid disease (9). Some studies have shown that there is an increased level of serum TPOab in women with PCOS compared to women without PCOS (3, 7). However, the included study populations are small.

Besides PCOS, hyperprolactinemia (HPRL) is also a common endocrine disorder in women of reproductive age which could result in anovulation. A population based cohort study observed a prevalence of HRPL of around 4% in female blood donors with a mean age of 30 years (10). It is often claimed that there is an association between PCOS and hyperprolactinemia, due to a common pathophysiological link. One of the hypotheses is that an acceleration of GnRH pulsatility results in a decrease in dopaminergic tone which causes increased levels of LH (which is often the case in PCOS) and an increase in prolactin levels (11, 12). The study of Hayashida et al. found that elevated levels of prolactin, coupled with the presence of macroprolactin, are not uncommon in women with PCOS and therefore important to determine (13). However, the literature addressing hyperprolactinemia in women with PCOS was found to be inconsistent (10, 13). The majority of studies included only a small number of women with PCOS and the definitions used to diagnose PCOS differed considerably. In order to determine whether a woman has PCOS in addition to hyperprolactinemia, prolactin levels have to be normalized before the diagnosis PCOS can be made. This can prove to be challenging. Therefore, the co-occurrence of hyperprolactinemia in women with PCOS is still unclear.

The recent international PCOS guideline could not give any recommendations regarding thyroid function and hyperprolactinemia in PCOS due to lack of sufficiently powered studies (14). Therefore, our aim was to determine the prevalence and future risk of thyroid dysfunction in a large group of women with PCOS diagnosed according to the Rotterdam criteria compared to a control group from the general population. Our second aim was to compare the prevalence of hyperprolactinemia between both groups. Furthermore, we also compared the prevalence of thyroid problems and hyperprolactinemia within the different PCOS phenotypes.

## Materials and methods

### Study population

All patients of the division of Reproductive Medicine (department of Obstetrics and Gynecology) of the Erasmus Medical Centre Rotterdam with cycle irregularities or clinical signs of hyperandrogenism, such as hirsutism or acne underwent a standardized endocrinological screening. This screening included a questionnaire, anthropometric measurements, hormonal evaluation and a transvaginal ultrasonography. Based on this screening, when they met the Rotterdam criteria, women were diagnosed with PCOS. According to these criteria, at least two of the three following symptoms must be present (1): oligo- or anovulation, (2) clinical and/or biochemical hyperandrogenism and (3) the presence of polycystic ovarian morphology (PCOM) (determined by a transvaginal ultrasound). It defines the following 4 phenotypes: phenotype A (oligo- or anovulation, hyperandrogenism and PCOM), phenotype B (oligo- or anovulation and hyperandrogenism), phenotype C (hyperandrogenism and PCOM), and phenotype D (oligo- or anovulation and PCOM). Other etiologies must be excluded (including thyroid disease, hyperprolactinemia, Cushing’s syndrome or androgen-secreting tumors) (15). In our cohort, we only included women with PCOS diagnosis, if PCOS characteristics remained after normalization of thyroid levels and prolactin levels when necessary. PCOM was characterized by the presence of 12 or more antral follicles (when an ultrasound was used with a transducer frequency of < 8 MHz) or 20 or more (when an ultrasound was used with a transducer frequency of > 8 MHz) in one or both ovaries, and/or increased ovarian volume (>10 cm^3^). Before the 20^th^ of August 2012, RIA (Siemens DPC, Los Angeles, USA) was used to measure testosterone. Biochemical hyperandrogenism was defined as a Free Androgen Index (FAI: (T*100/SHBG)) > 4.5 and/or a testosterone value ≥ 3 nmol/L (16). After the 20^th^ August 2012 testosterone was measured with liquid chromatography-tandem mass spectrometry, and therefore we used a FAI cut-off > 2.9 and a total testosterone cut-off > 2.0 nmol/L. For clinical hyperandrogenism we used the modified Ferriman-Gallwey score, with a cut-off value ≥ 5 (14). In a large group of women with PCOS who underwent the standardized endocrinological screening between 1993 and 2009, besides TSH and FT4, thyroid antibodies were also measured. These women were included in the study.

The control group consisted of women who participated in the HAVEN study, a Dutch acronym for the study of heart anomalies and the role of genetic and nutritional factors (17). This study was a case-control family study of which the inclusion took place from June 2003 to January 2010 at the department of Obstetrics and Gynecology of Erasmus MC, University Medical Centre in Rotterdam. The rationale and design of the HAVEN study have been published previously (17). In short, the aim of this study was to investigate determinants in the pathogenesis and prevention of congenital heart disease in offspring of otherwise healthy mothers. They investigated children with congenital heart disease and both their parents as well as their healthy siblings. All these mothers were included in our study and had regular menstrual cycles, conceived spontaneously and had no reported medical history with PCOS.

### Hormone and autoantibody measurements

Blood samples were obtained during the standardized endocrinological screening for women with PCOS. In the control group TSH, FT4 and TPOab were measured in serum that was stored at -20 degrees Celsius. Hormones were measured in the Diagnostic Laboratory Endocrinology and TPOab in the Laboratory Medical Immunology, both part of the Erasmus University Medical Centre.

TSH levels were measured on the Immulite platform (Siemens, Diagnostic Products Corporation, Breda, the Netherlands) in the PCOS group and by Lumipulse G1200 (Fujirebio, Barcelona, Spain) in the control group. These values were compared without a conversion formula, because there was no calibration bias. According to our laboratory, normal serum TSH levels were between 0.56 - 4.27 mIU/L. To compare TSH values between both groups we excluded women in these analyses who used medication to treat thyroid dysfunction. FT4 levels were measured by Ortho Vitros ECiQ in the PCOS group and by Lumipulse G1200 (Fujirebio, Barcelona, Spain) in the control group. Therefore, we converted the FT4 values of the PCOS women, using the following formula: FT4 (Lumipulse) = FT4 (Ortho) * 0.94 + 1.4. According to our laboratory, normal serum FT4 levels were between 13.5-24.3 pmol/L. To compare FT4 levels between both groups we excluded women in these analyses who used medication to treat thyroid dysfunction. To compare the prevalence of thyroid dysfunction we did not exclude women who used thyroid medication. Women were considered as having hypothyroidism if 1) they were already diagnosed with hypothyroidism and used medication or 2) had a TSH value > 4.27 mIU/L and a FT4 < 13.5 pmol/L. If TSH values were >4.27 mIU/L but FT4 was within the normal range (13.5 – 24.3 pmol/L) they were labelled as having subclinical hypothyroidism. Women were considered as having hyperthyroidism if 1) they were already diagnosed with hyperthyroidism and used medication or 2) had a low TSH value (<0.56 mIU/L) and had high FT4 value (>24.3 pmol/L).

TPOab were measured with fluorescence enzyme-immuno assay (FEIA) using the Phadia 250 analyzer (Thermo Fisher Scientific, Freiburg, Germany), according to manufacturer’s instructions. In the PCOS group The ImmunoCAP™ TPO method was employed and in the control group the EliA™ TPO method (both Thermo Fisher Scientific). Due to different analysis methods other cut-off values were used for both groups. For the PCOS group TPOab <60 iU/ml was considered as negative, 60-100 iU/ml as dubious and >100 iU/ml as positive. For the control women TPOab < 25 iU/ml was considered as negative, 25.0-35.0 iU/ml as dubious and > 35.0 iU/ml as positive.

Prolactin was measured by Lumipulse G1200 (Fujirebio, Barcelona, Spain) or by Immulite platform 2000XPi (Siemens Diagnostic Products Corporation, Munich, Germany). Hyperprolactinemia was considered by prolactin levels > 0.98 U/L when measured by Immulite and > 0.7 U/L when measured by Lumipulse.

### Statistical analysis

The baseline clinical and endocrine characteristics are presented as numbers with percentages and as medians with interquartile ranges (IQR). To compare continuous data, we used the Mann-Whitney U test (non-parametric). To compare dichotomous variables, such as the prevalence of thyroid diseases we used a Chi-square test or a Fisher exact test (when n <5 in one or more cells). To adjust for age and BMI, we used linear regression models. TSH was not normally distributed and was log-transformed before linear regression analysis. To examine whether there was a statistically significant difference between the medians of the four phenotypes, we performed a Kruskal Wallis test and used a Bonferroni correction for multiple comparisons. A P-value <0.05 was considered statistically sign ficant.

## Results

We included 1429 women with PCOS and 299 controls. **Table 1** shows the baseline characteristics. The median age of women with PCOS was significantly lower than the median age of the control group (28.2 years (IQR 24.6 – 31.7) versus 32.8 years (IQR 29.7 – 35.8); P < 0.01). As expected, the median BMI of women with PCOS was significantly higher than the BMI of controls (25.5 kg/m^2^ (IQR 21.9 – 30.7) versus 24.4 kg/m^2^ (IQR 22.3 – 27.5); P < 0.01). Furthermore, in the control group more women had a Caucasian ethnicity than in the PCOS group (85.3% versus 61.3%; P < 0.01). All women in the control group had a regular cycle compared to 1.9% in the PCOS group.

**Table 1:** Baseline characteristics: PCOS group and the control group.

	PCOS (n=1429)	Control group (n=299)	P-value
**Age**	28.2 (24.6-31.7)	32.8 (29.7-35.8)	<0.01
**BMI (kg/m^2^)**	25.5 (21.9-30.7)	24.4 (22.3-27.5)	<0.01
**Ethnicity** ** Caucasian** ** Non-Caucasian**	868 (61.3%) 548 (38.7%)	255 (85.3%) 44 (14.7%)	<0.01
**Cycle** ** Regular** ** Amenorrhea** ** Oligomenorrhea**	27 (1.9%) 383 (26.8%) 1019 (71.3%)	299 (100%) 0 (0%) 0 (0%)	<0.01
**Smoking at this moment** **No** **Yes**	703 (76.2%) 220 (23.8%)	191 (78.9%) 51 (21.1%)	0.37

Data are presented as medians with interquartile ranges or as numbers with percentages. BMI, body mass index.

### Thyroid dysfunction

**Table 2** shows the prevalence of thyroid diseases in the PCOS group and control group. No differences in prevalence of hypothyroidism (1.9% versus 2.7%; P = 0.36), subclinical hypothyroidism (3.2% versus 2.7%; P = 0.64), and hyperthyroidism (0.5% versus 0%; P = 0.99) were found between the groups. After excluding 31 women who used thyroid medication, there was no difference in serum TSH levels between the PCOS group and control group (1.55 mIU/L versus 1.48 mIU/L; P = 0.49). The serum FT4 values, although still within the normal range, were higher in the PCOS group (18.1 pmol/L versus 17.7 pmol/L; P < 0.01), also after adjusting for age and BMI (P < 0.05). After adjusting for age and BMI there was no difference in the frequency of positive TPOab between cases and controls (5.7% versus 8.7%; P = 0.12). Additionally, we performed a subanalysis on 235 women with PCOS and 235 age- and BMI-matched controls (**Supplementary Table 1**) (18). In brief, we found a similar prevalence in thyroid diseases and TPOab in women with PCOS compared to controls (all P-values above 0.05). Also, levels of TSH and FT4 were similar between both groups (P = 0.47 and P = 0.84 respectively).

**Table 2:** Thyroid dysfunction and hyperprolactinemia in women with PCOS and controls.

	PCOS (n=1429)	Control group (n=299)	P-value	Adjusted P-value
**Thyroid diseases** ** None** ** Hypothyroidism** ** Subclinical hypothyroidism** ** Hyperthyroidism**	1324 (94.4%) 26 (1.9%) 45 (3.2%) 7 (0.5%)	282 (94.6%) 8 (2.7%) 8 (2.7%) 0 (0%)	0.89 0.36 0.64 0.99	0.60 0.40 0.59 0.99
**TSH (mIU/L)**	1.55 (1.07-2.21)	1.48 (1.08-2.20)	0.49	0.54
**TPOab** ** Positive** ** Negative**	81 (5.7%) 1347 (94.3%)	26 (8.7%) 273 (91.3%)	0.05	0.12
**FT4 (pmol/L)**	18.1 (16.3-20.2)	17.7 (16.3-19.3)	<0.01	<0.05
**Hyperprolactinemia** ** Yes** ** No**	15* (1.1%) 1414 (99.0%)	9 (3.0%) 289 (97.0%)	0.01	0.05

Data are presented as medians with interquartile ranges or as numbers with percentages. The adjusted P-value is adjusted for BMI and age. Only to compare TSH and FT4 levels, women who used thyroid medication (n=31) were excluded. TPOab were seen as positive in the PCOS group when >100 U/ml and in the control group when >35 U/ml. *5 of these 15 women had a pituitary abnormality, like a macroprolactinoma. TPOab, anti-thyroid peroxidase antibodies; FT4, Free Thyroxine.

### Hyperprolactinemia

The prevalence of hyperprolactinemia was similar in women with PCOS compared to controls (n=15 (1.1%) versus n=9 (3.0%); P = 0.05), after adjusting for age and BMI (**Table 2**). Five PCOS patients with hyperprolactinemia had pituitary abnormalities, including (micro)prolactinoma and microadenoma, in the control group there were no women with pituitary abnormalities. Additional analysis on 235 women with PCOS and 235 age- and BMI-matched controls, showed a similar prevalence of hyperprolactinemia (3/235 (1.3%) versus 7/235 (3.0%); P = 0.20) (**Supplementary Table 1**).

### PCOS phenotype related to thyroid function and prolactin levels

After dividing all PCOS women into the different phenotypes, according to the Rotterdam criteria, 741 (51.9%) women had phenotype A, 95 (6.6%) women had phenotype B, 30 (2.1%) women had phenotype C, 444 (31.1%) women had phenotype D. Of 119 women some data was missing to classify the precise phenotype. BMI was significantly different between all four phenotypes: 27.3 kg/m^2^ (IQR 23.1 – 32.2) in phenotype A, 30.4 kg/m^2^ (IQR 26.2 – 35.0) in phenotype B, 28.4 kg/m^2^ (IQR 24.9 – 32.4) in phenotype C, and 22.5 kg/m^2^ (IQR 20.3 – 25.5) in phenotype D, with an overall P-value < 0.01.

**Figure 1:**
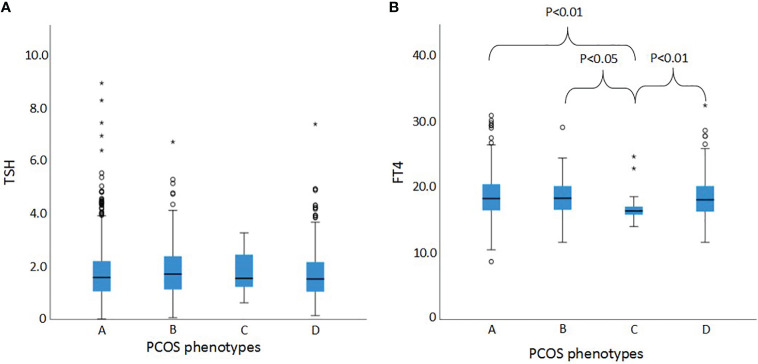
TSH and FT4 levels of the different PCOS phenotypes **(A)** TSH levels in serum of women with PCOS divided and compared between the four phenotypes. **(B)** FT4 levels in serum of women with PCOS divided and compared between the four phenotypes. 24 women were excluded, only for comparing TSH and FT4 levels,because of thyroid medication use. TSH, thyroid-stimulating hormone; FT4, Free Thyroxine; Phenotype A, hyperandrogenism + ovulatory dysfunction + polycystic ovaries; Phenotype B, hyperandrogenism + ovulatory dysfunction; Phenotype C, hyperandrogenism + polycystic ovaries; Phenotype D, ovulatory dysfunction + polycystic ovaries. The dots and the stars in this figure mean outliers. Dots mean outliers including the formula: IQR * 1.5. The stars mean far outliers, including the formula: IQR * 3.0. This is a setting in SPSS by default.

**Figure 2:**
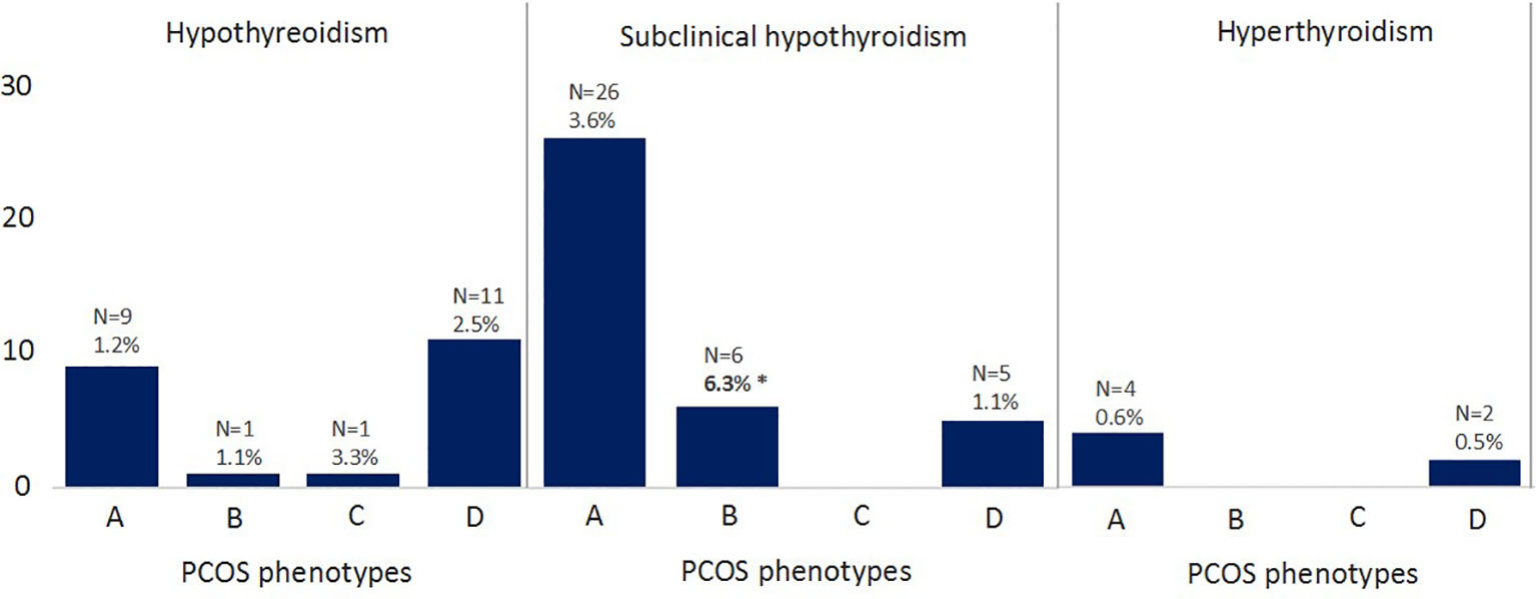
Frequency of thyroid diseases in the PCOS phenotypes Phenotype A, hyperandrogenism + ovulatory dysfunction + polycystic ovaries; Phenotype B, hyperandrogenism + ovulatory dysfunction; Phenotype C, hyperandrogenism + polycystic ovaries; Phenotype D, ovulatory dysfunction + polycystic ovaries. *Phenotype B had significantly higher prevalence of subclinical hypothyroidism compared to the other phenotypes (P < 0.05).

**Figure 1** shows the TSH levels and FT4 levels in serum of women with PCOS divided in the different phenotypes. There were no differences in TSH levels between the phenotypes (**Figure 1A**). FT4 levels of phenotype C were significantly lower compared to the three other phenotypes (**Figure 1B**), but still within the normal range (16.3 pmol/L (IQR 15.8-17.4)). **Figure 2** shows the prevalence of hypothyroidism, subclinical hypothyroidism and hyperthyroidism in the different phenotypes of PCOS. Only subclinical hypothyroidism was significantly different between the phenotypes, in which phenotype B has the highest prevalence (6.3%), compared to phenotype A (3.6%) and phenotype D (1.1%). The prevalence of hyperprolactinemia was similar between the phenotypes (5/736 (0.7%) in phenotype A, n= 0 in phenotype B, 1/29 (3.4%) in phenotype C, and 9/435 (2.1%) in phenotype D, P = 0.08).

We also compared women with a BMI < 25 kg/m^2^ (normal BMI) with PCOS women with a BMI ≥ 25 kg/m^2^ (overweight or obese). Median TSH levels were higher in women with a BMI ≥25 kg/m^2^ compared to women with a normal BMI (1.62 mIU/L versus 1.47 mIU/L; P < 0.01). Also, the prevalence of subclinical hypothyroidism was higher in women with a BMI ≥25 kg/m^2^ (4.5% versus 1.8%; P < 0.05). There were no differences in FT4 levels, the prevalence of hypothyroidism, the prevalence of hyperthyroidism, and in the prevalence of positive TPOab between the two groups. The prevalence of hyperprolactinemia was higher in the lean group compared to the obese group (n=13 (1.9%) versus n=2 (0.3%); P < 0.01).

## Discussion

This study shows that the prevalence of thyroid diseases, serum TSH levels, the prevalence of TPOab, and the prevalence of hyperprolactinemia are similar in women with PCOS compared to controls. FT4 serum levels were slightly higher in women with PCOS compared to controls, but still well within the normal range.

We found a similar prevalence of hypothyroidism (around 2%) as in a previously published study from Glintborg et al. (19). This was a register-based study which included three groups: 18,476 women who had a PCOS or hirsutism diagnosis in a Danish register, a PCOS cohort of 1146 women from a Danish Hospital, and 54,757 controls from the civil population register. In this study, however, a higher prevalence of hyperthyroidism was found and therefore a higher prevalence of total event rate of all thyroid diseases (9% and 7%) when compared to our results (5.6%). Furthermore, a lower total event rate of thyroid diseases in controls (3% versus 5.4% in our control group) was reported, this might explain their result of a significant difference in the prevalence of thyroid disorders in women with a registered PCOS or hirsutism diagnosis than in controls. These contradictory results might also be due to the fact that we included a hospital based control population and they included a civil population register cohort. The inclusion of women with a PCOS or hirsutism ICD10 diagnosis and a control group from a population register, might cause potential selection bias, due to the fact that all women with PCOS visited a hospital at least once and therefore were more thoroughly screened for thyroid dysfunction compared to controls. Moreover, they also included women only diagnosed with hirsutism, which might not have PCOS at all. This could have resulted to a phenotypically different PCOS population and thus result in a different outcome. No data was available on PCOS phenotype in this study. To assess whether the future risk of thyroid dysfunction is increased in women with PCOS, we assessed the prevalence of positive TPOab. We found a similar prevalence of positive TPOab in women with PCOS compared to controls, which suggests a similar future risk for thyroid dysfunction. Indeed, a follow-up study, including older women with PCOS (around the age of 70) and age matched controls, found similar TSH levels and TPOab levels (20). This study even found a lower prevalence of hypothyroidism in women with PCOS. In the same population 11 years later, similar TSH and FT4 levels were assessed in women with PCOS compared to controls (both groups around the age of 80) (21). Also when including a larger control group with males, no differences in TPOab were found between these groups (22). The authors suggest that androgens might play a protecting role in developing hypothyroidism, since men with low androgens and women with Turner syndrome (with low androgens as well) both have a higher prevalence of hypothyroidism. However, in our subgroup analysis, when comparing different phenotypes, we only found a higher prevalence in phenotype B for subclinical hypothyroidism compared to phenotype A and D, which is not explained by different androgenic status. In contrast, phenotype B had the lowest prevalence of positive TPOab. The low number of women with thyroid diseases in each phenotype subgroup in the current study, might also explain why we did not observe such differences between the groups. Therefore, these results should be interpreted with caution.

In our subanalysis we found increased levels of TSH and an increased prevalence of subclinical hypothyroidism in women with PCOS and a BMI < 25 kg/m^2^ than in women with PCOS and a BMI ≥ 25 kg/m^2^. This is in line with the literature, which states that BMI is positively associated with TSH levels (23, 24). This strongly suggests that BMI is a significantly more important risk factor for developing thyroid dysfunction than PCOS itself. This emphasizes the importance of adequately educating women with PCOS about a healthy lifestyle, considering that women with PCOS are more prone to experiencing overweight or obesity.

In addition to thyroid dysfunction, there is an ongoing debate about whether there is an association between PCOS and HPRL, since hyperprolactinemia should be ruled out in advance, before the diagnosis of PCOS can be made with use of consensus criteria (11). A retrospective study, that diagnosed 528 women with PCOS by medical records, showed elevated prolactin levels in 11.4% of which 43.2% of these women had pituitary adenomas (25). In another study 5.8% of the women with PCOS (n=227) had elevated prolactin levels and in all of these women, macroprolactin, which is an inactive form of prolactin, was detected (13). The prevalence of HPRL in our study is similar (7%), and only a few women had pituitary abnormalities, including prolactinomas. The study of Mahboobifard et al., in which the results showed higher serum prolactin levels in women with PCOS (aged ≤35 years) compared to health controls, substantiate the underlying pathway of a lower dopaminergic control. This causes an increase in LH and prolactin levels (11, 26). We also found a weak and positive correlation (Pearson’s r of 0.073; P < 0.01) between LH levels and prolactin levels in the PCOS group (data not shown). However, Mahboobifard et al. also showed that these differences disappeared after the age of 35.

Since oral contraceptive pills (OCP) can influence prolactin levels, we compared the prolactin levels within the PCOS group, between women who used or did not use OCP’s. No difference was found between those who used OCP’s and those with PCOS who did not. No data was available about OCP use in the control group, which could have influenced the outcomes concerning prolactin levels. However, the prevalence of hyperprolactinemia would have remained almost the same even if we had adjusted for this, as OCP only minimally increase prolactin levels (27).

Selection bias is a potential limitation of our study, because we may have underestimated the real prevalence of thyroid dysfunction or hyperprolactinemia in the general population, as these conditions are often associated with ovulatory dysfunction and fertility problems. However, the medical history of the control participants was extensively documented. Therefore, women who have experienced thyroid or pituitary problems in the past, but in whom this did not influence the fertility during the periconception period, were also included in our study. Furthermore, this is the first study with an extensive well-phenotyped PCOS group, in which multiple other endocrine problems were excluded.

In conclusion, the prevalence of thyroid disorders and the risk of developing thyroid dysfunction appears to be the same in women with PCOS compared to the general population. Additionally, we found a similar prevalence of hyperprolactinemia in women with PCOS compared to controls.

## Data availability statement

The raw data supporting the conclusions of this article will be made available by the authors, without undue reservation.

## Ethics statement

The studies involving humans were approved by The Erasmus MC Medical Ethics Committee. The studies were conducted in accordance with the local legislation and institutional requirements. The human samples used in this study were acquired from primarily isolated as part of your previous study for which ethical approval was obtained. Written informed consent for participation was not required from the participants or the participants’ legal guardians/next of kin in accordance with the national legislation and institutional requirements.

## Author contributions

JL, YL, KvdH, and KS contributed to conception and design of the study. MS and RW performed the laboratory analysis. KS and KvdH performed the descriptive analysis and drafted the manuscript. All authors contributed to the article and approved the submitted version.
