# Supplementary material for: The prevalence of thyroid dysfunction and hyperprolactinemia in women with PCOS
Source: Front Endocrinol (Lausanne). 2023 Oct 3;14:1245106. doi: 10.3389/fendo.2023.1245106 (PMC10579902; doi:10.3389/fendo.2023.1245106)
Supplement: Supplementary file 1 [file Table_1.docx]

Supplementary Material

The prevalence of thyroid dysfunction and hyperprolactinemia in women with PCOS

**Kim van der Ham^1^*†, Karlijn J Stekelenburg^1^†, Yvonne V Louwers^1^, Wendy van Dorp^1^, Marco W J Schreurs^2^, Ronald van der Wal^3^, Régine P M Steegers-Theunissen^4^, Joop S E Laven^1^**

***Correspondence:** K. van der Ham, MD, Division of Reproductive Endocrinology and Infertility, Department of Obstetrics and Gynecology, Erasmus University Medical Centre, Dr. Molewaterplein 40, 3015 GD, Rotterdam, The Netherlands. E-mail: [k.vanderham@erasmusmc.nl](mailto:k.vanderham@erasmusmc.nl), ORCID ID 0000-0003-3401-4585.

|  | **PCOS**  **(n=235)** | **Control group**  **(n=235)** | **P-value** |
| --- | --- | --- | --- |
| **Age** | 31.7 (28.8 – 34.4) | 31.8 (28.7 – 34.4) | 0.96 |
| **BMI (kg/m^2^)** | 24.5 (22.1 – 27.4) | 24.4 (22.1 – 27.6) | 0.97 |
| **Thyroid diseases  None  Hypothyroidism  Subclinical hypothyroidism  Hyperthyroidism** | 215 (91.5%)  5 (2.1%)  7 (3.0%)  1 (0.4%) | 222 (94.5%)  5 (2.1%)  7 (3.0%)  0 (0%) | 0.79  0.61  0.96  0.49 |
| **TSH (mIU/L)** | 1.59 (1.07 – 2.33) | 1.49 (1.09 – 2.12) | 0.47 |
| **TPOab  Positive**  **Negative** | 14 (6.0%)  221 (94.0%) | 23 (9.8%)  212 (90.2%) | 0.12 |
| **FT4 (pmol/L)** | 17.8 (15.9 – 20.1) | 17.7 (16.2 – 19.3) | 0.84 |
| **Hyperprolactinemia  Yes  No** | 3* (1.3%)  232 (98.7%) | 7 (3.0%)  228 (97.0%) | 0.20 |

**Supplemental Table 1.** Thyroid dysfunction and hyperprolactinemia in women with PCOS and age- and BMI-matched controls

Data are presented as medians with interquartile ranges or as numbers with percentages. To compare TSH and FT4 levels, women who used thyroid medication (n=9) were excluded. TPOab were seen as positive in the PCOS group when >100 U/ml and in the control group when >35 U/ml. *One of these three women had a pituitary abnormality, like a macroprolactinoma. TPOab, anti-thyroid peroxidase antibodies; FT4, Free Thyroxine.
